# Phase transition in multimode nonlinear parity-time-symmetric waveguide couplers

**DOI:** 10.1038/srep19826

**Published:** 2016-02-02

**Authors:** Wiktor Walasik, Natalia M. Litchinitser

**Affiliations:** 1 Department of Electrical Engineering, University at Buffalo, The State University of New York, Buffalo, New York 14260, USA

## Abstract

Parity-time-symmetric (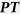-symmetric) optical waveguide couplers offer new possibilities for fast, ultracompact, configurable, all-optical signal processing. Here, we study nonlinear properties of finite-size multimode 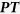-symmetric couplers and predict the nonlinear oscillatory dynamics that can be controlled by three parameters: input light intensity, gain and loss amplitude, and input beam profile. Moreover, we show that this dynamics is driven by a 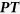 transition triggered by nonlinearity in these structures, and we demonstrate that with the increase of the number of dimers in the system, the transition threshold decreases and converges to the value corresponding to an infinite array. Finally, we present a variety of periodic intensity patterns that can be formed in these couplers depending on the initial excitation.

Quantum mechanical observables correspond to operators with a real spectrum of eigenvalues^1^. Hermitian operators are the most well-known class of such operators. However, a more general class of non-Hermitian operators that possess a real spectrum was described by Bender *et al*.^2^,^3^ in 1998. These parity-time-symmetric (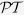-symmetric) operators commute with a subsequent action of the parity operator 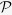 and the time inversion operator 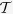. Consequently, 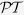-symmetric Hamiltonians have real spectra and describe physical phenomena, provided that the complex potentials *V* fulfill the condition 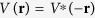. Optical systems, where the light propagation is described by the nonlinear Schrödinger equation, offer an efficient platform for implementation of 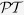 symmetry. The complex dielectric permittivity distribution *ϵ*(**r**) plays the role of potential and the imaginary part of the permittivity corresponds to gain or loss^4^,^5^.

The optical 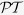 symmetry in the linear regime was experimentally demonstrated in waveguide couplers^6^, which have a critical importance for the future ultracompact, fast, and configurable all-optical switching. In the nonlinear regime, optical systems with gain and loss were studied^7^,^8^,^9^, where suppression of time reversal^10^ and unidirectionality^11^ were demonstrated. Phenomena related to nonlinear 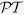 symmetry were also investigated in periodic systems, where solitons^12^,^13^,^14^,^15^,^16^,^17^, breathers^18^ and their stability^19^,^20^,^21^ were analyzed.

For fixed geometrical parameters, 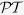-symmetric systems can be in the full or the broken 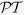-symmetric regime depending on the ratio between the imaginary *ϵ*_IM_ and the real part *ϵ*_RE_ of the permittivity modulation depth (*ϵ*_IM_/*ϵ*_RE_). For low values of this ratio, the system is in the full 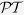-symmetric regime and has purely real eigenvalues^6^,^22^. By increasing the *ϵ*_IM_/*ϵ*_RE_ ratio, the 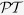 transition threshold is reached, and the system transforms into the broken 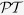-symmetric regime, where a pair of modes (one with gain and one with loss) has complex conjugate effective indices. In the linear regime, the *ϵ*_IM_/*ϵ*_RE_ ratio is typically changed by varying *ϵ*_IM_, responsible for gain and loss in the system. However, changes of the real part of the permittivity also influence the ratio of *ϵ*_IM_/*ϵ*_RE_. The amplitude of *ϵ*_RE_ in nonlinear systems can be controlled by varying the incident light intensity. Recently, a nonlinearity-triggered transition from the full to the broken 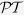-symmetric regime (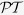 transition) was reported in an infinite periodic array of waveguides described by cosine-like permittivity distribution^23^.

In this paper, we study the nonlinear, 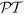-symmetric, ultracompact couplers consisting of a few cosine-like waveguide dimers that can be readily integrated on a chip. Here, the term dimer refers to a single optical waveguide with one loss and one gain region. Figure 1 shows the three-dimer geometry under investigation. The goal of this work was to study how a combination of nonlinearity and 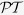-symmetry breaking condition in multimode couplers can be used to control the dynamics of beam propagation in finite waveguide arrays. We report a new type of linear dependency of modal effective-index on the imaginary part of permittivity modification depth for a multimode 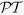-symmetric coupler^24^ built of more than one dimer. We predict a 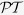 transition in such systems and study the dependence of the transition threshold on the number of dimers in the coupler. We demonstrate that a variety of periodic patterns self-repeating in the direction of propagation can be induced by controlling either the input light intensity, gain/loss amplitude, or the input beam profile. These findings may offer new functionalities in the design of ultracompact, configurable all-optical devices incorporated in on-chip photonic systems.

Light propagation in one-dimensional (1D) 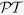-symmetric structures with cubic nonlinearity is studied using a scalar wave equation for the electric field *E*(*x*, *z*):


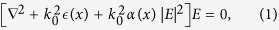


where *k*_0_ = 2*π*/*λ* is the free-space wavevector, *λ* is the free-space wavelength of light, and the operator 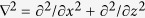 denotes the 2D Laplacian. The light propagates along the *z*-direction, and both the structure and the field distributions are assumed to be invariant along the *y*-direction. The linear complex relative permittivity distribution is described by 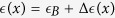, where *ϵ*_*B*_ denotes the background relative permittivity and Δ*ϵ*(*x*) describes the linear complex permittivity modulation depth. The Kerr-type nonlinearity strength is quantified by the parameter *α*. The nonlinearity *α*(*x*) is nonzero only inside of the waveguides.

Using the slowly varying envelope approximation 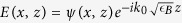, Eq. (1) is transformed into the (1 + 1)D nonlinear Schrödinger equation


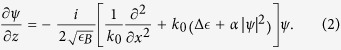


In order to analyze the nonlinear dynamics of the light propagation in 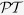-symmetric waveguides, we solve Eq. (2) using the split-step Fourier method^25^,^26^.

Previously, nonlinear 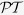 transition was theoretically studied in an infinite periodic cosine-like waveguide array^23^. Here, we analyze simpler, finite-size systems of waveguides, where we find a rich variety of nonlinear phenomena. Let us consider a finite-size array built of three dimers (shown in Fig. 1) described by


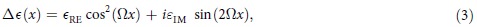


where 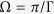, Γ denotes the full width of the dimer, and the structure parameters are the following: Γ = 3 *μ*m, *ϵ*_RE_ = 0.05, and *α* = 10^−19^ m^2^/V^2^ at *λ* = 0.63 *μ*m.

Let’s first consider a linear system 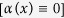. The field profiles that propagate with an effective index *n*_eff_ without changing their shape are sought in the form 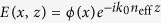. This allows us to transform Eq. (1) into an eigenvalue problem for the modes of the waveguide described by *ϵ*(*x*). The eigenvalue problem is solved using a finite-difference method resulting in the dependency of the modal effective-index on the imaginary part of permittivity modification depth [shown in Fig. 2(a,b)] and the corresponding field profiles of the eigenmodes [shown in Fig. 2(c,d)]. We use these diagrams to locate the 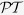 transition threshold. For the parameters of the dimers chosen here, the waveguide supports more than one mode. For low values of *ϵ*_IM_, there exist four modes with purely real effective indices. With the increase of *ϵ*_IM_, two new modes emerge at 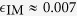 and 0.034. Meanwhile, the pairs of modes with real effective indices undergo a transition to the broken 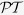-symmetric regime. For high values of *ϵ*_IM_, the lower-order conjugated mode pairs vanish, leaving only the highest-order mode pair, in which one mode experiences gain and the other experiences loss during the propagation.

In a single multimode dimer [Fig. S1(a) in supplementary materials], the curves corresponding to the effective index of higher-order mode pairs appear below the curves of the two first modes (see, Fig. S2 in supplementary materials and Figs 2, 5 and 7 in ref. 24). On the contrary, in the case of a multimode array built of multiple dimers, the curves corresponding to the effective index of mode pairs with purely real effective indices do not lay above each other but inside (curves corresponding to the higher-order modes are enclosed by these of the lower-order mode pairs), as it is seen in Fig. 2. Above the 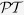 transition, the curves corresponding to 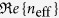 of the two higher-order modes intersect with the curve of one of the lossless lower-order modes [see e.g., the point labeled *A* in Fig. 2(a) corresponding to *ϵ*_IM_ = 0.0314]. At these points, three modes with the same value of 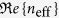 exist simultaneously in the dimer array. One of them experiences gain, one loss, and one propagates without change of its amplitude.

The diagrams presented in Fig. 2 provide the value of *ϵ*_IM_ where the first (for lowest value of *ϵ*_IM_) 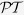 threshold occurs in the system. For the array of three dimers, this value is *ϵ*_IM_ = 0.0268. Below the threshold we find five symmetric (with respect to the center of the array *x* = 0) lossles modes with real effective indices. Above the threshold, in addition to the three lossless modes, we find two modes: one for which the field profile is shifted slightly to the gain region (gain mode) and one shifted to the loss region (loss mode). The corresponding field profiles are presented in Fig. 2(c,d).

In addition to the coupler built of three dimers studied here, we have analyzed the 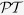 transition in structures made of different numbers of dimers. Here, we summarize the values of the 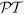 transition threshold 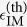 obtained: for a single dimer 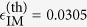
27; for two dimers: 0.0278 (see supplementary materials for more details), and for three dimers: 0.0268. Additional calculations show that for the case of four dimers, this value is equal to 0.0262. This suggests two conclusions. Firstly, the threshold value for a finite-size array of dimers is higher than the threshold for the infinite array of dimers described by Eq. (3), which was reported in refs 12,23,28 to be 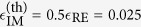. This can be understood considering a simple model based on the fact that light concentrates in the regions with high permittivity. Then, the rate with which the overlap integral between a test field profile and the permittivity profile decreases, while shifting the test field profile along the *x*-direction quantifies how difficult it is to break the symmetry in the system and induce the 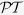 transition. The higher the number of dimers, the slower the change of this overlap integral is and, consequently, the easier it is to induce the 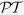-symmetry-breaking transition. The dependency of the threshold 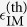 as a function of the number of dimers *N* can be fitted as 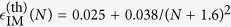. This result is in agreement with the 1/*N*^2^ dependency for the 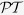-symmetric lattices predicted in refs 29,30, but it has a off-set value for infinite periodic lattice. This means that the 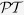-symmetric system studied here does not become threshold-less in the limit of infinite periodic lattice^23^,^31^,^32^. The second conclusion states that with the increase of the number of dimers, the threshold value converges to the value corresponding to the infinite array as expected because the structure becomes more similar to the infinite array.

Figure 3 presents the evolution of the light propagating in the nonlinear coupler built of three 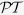-symmetric dimers described by Eq. (3). Initially, the system is in the broken 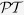-symmetric regime, and the light is injected into the gain mode of the system. The intensity of this mode grows rapidly, causing the nonlinear increase of the real part of permittivity, which transforms the system to the full 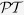-symmetric regime. In this regime, the light is attracted toward the center of each dimer (where the permittivity is the highest), and due to the transverse momentum, light crosses the center and is mostly located in the loss region^22^. Consequently, the light intensity and the nonlinear modification of permittivity decrease bringing the system back to the broken 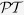-symmetric regime. This cycle repeats as shown in Fig. 3(a) and the evolution of the *x*-coordinate of the center of mass (COM) of the intensity distribution 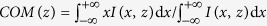 presented in Fig. 3(b). For the input beam energy chosen in Fig. 3(a,b), the total power in the system increases 25 times during one cycle, and the period of the cycle is approximately equal to 1 mm. Lowering the input power results in an increase of the propagation length required for the power to grow and initiate the oscillations, but the oscillation period and the maximum power remains approximately the same. On the contrary, an increase of the input power results in a decrease of the oscillation period (down to 0.5 mm) and an increase (1.5 times) of the peak power [see Fig. 3(c,d)].

Figure 4 shows nonlinear propagation of light in the multimode system studied in Figs 2 and 3, but this time instead of exciting a single (gain) mode, we excite a mixture of modes. Figure 4(a) shows the result of excitation with a wide Gaussian beam illuminating all three dimers. This beam excites all the modes of the waveguide (except the antisymmetric mode 2), and these modes interfere during the propagation. This interference results in a doubly periodic light distribution pattern. The high frequency oscillations result from the interference of all the modes that have different effective indices. Additionally, we observe low frequency oscillations, that are the result of the nonlinear 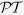 transition that couples light between the gain and the loss modes. The resulting pattern resembles the triangular lattice. A similar interference pattern is observed when a mixture of the fundamental mode and the gain mode are excited [see Fig. 4(b)].

On the contrary, the excitation of the mixture of mode 2 and the gain mode results in a different interference pattern, as shown in Fig. 4(c). Mode 2 is mainly localized in the side waveguides [see green curves in Fig. 2(a)]; therefore, the high frequency interference pattern is observed in these two waveguides only. The low frequency pattern attributed to the energy exchange between the gain and the loss mode is spread over all three waveguide dimers similar to earlier cases. Here, the pattern is less regular than before and consists of an elongated maximum in the middle waveguide and a few shorter maxima in the side waveguides.

In conclusion, we have studied nonlinear dynamics of light propagation in finite-size parity-time-symmetric couplers built of three dimers, and reported a new type of linear dependency *n*_eff_(*ϵ*_IM_) for multimode systems built of several dimers, where the curves corresponding to higher-order mode pairs are enclosed in the curves of lower-order mode pairs. This contrasts with the case of multimode single dimers, where the *n*_eff_(*ϵ*_IM_) curves of higher-order modes appear below the curves corresponding to lower-order modes. Moreover, the nonlinearly triggered transition from the full to the broken 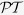-symmetric regime can be observed in such a system. The threshold for this 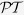 transition was found to be higher in finite-size systems than for infinite periodic potential. Finally, we predicted a variety of periodic patterns that can be formed in the multimode coupler by controlling the initial excitation conditions. These predictions may find potential applications in all-optical signal processing, high-speed opto-electronic circuits, and optical communication systems.

## Additional Information

**How to cite this article**: Walasik, W. and Litchinitser, N. M. Phase transition in multimode nonlinear parity-time-symmetric waveguide couplers. *Sci. Rep*. **6**, 19826; doi: 10.1038/srep19826 (2016).

## Supplementary Material

Supplementary Information

## Figures and Tables

**Figure 1:**
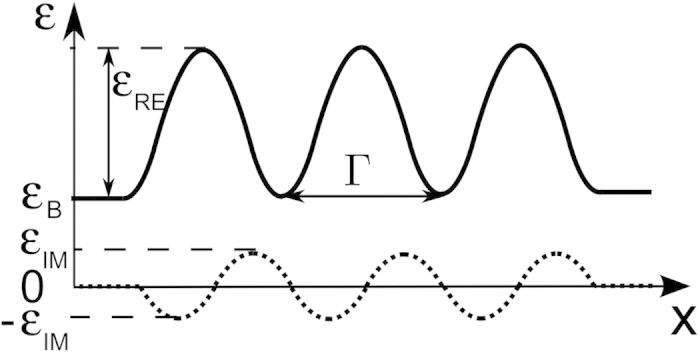
Geometry of the studied *PT*-symmetric coupler with its parameters. The size (period) of the cosine-like dimers is denoted by Γ. *ϵ*_*B*_ denotes the background relative permittivity; *ϵ*_RE_ and *ϵ*_IM_ denote the modulation amplitude of the real and the imaginary part of relative permittivity, respectively.

**Figure 2:**
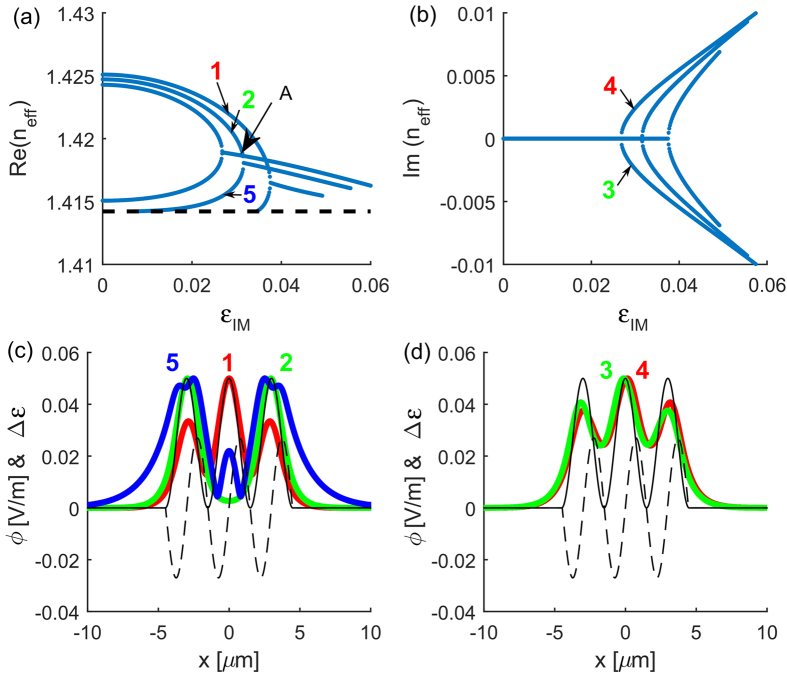
(**a**) The real and (**b**) the imaginary part of the effective index of the modes as a function of the imaginary part of permittivity *ϵ*_IM_ in the linear 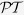-symmetric coupler composed of three dimers. The black dashed line denotes the refractive index of a background medium 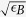. At point labeled *A* (for *ϵ*_IM_ = 0.0314) the curves corresponding to modes 2, 3, and 4 intersect. (**c**,**d**) Absolute value of the field distributions 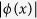 of (**c**) the lossless modes [mode 1 (fundamental)—red, mode 2—green, mode 5 (lowest effective index)—blue], (**d**) the coupled modes with loss (green) and gain (red) at *ϵ*_IM_ = 0.027. Black curves indicate 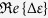 (solid) and 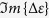 (dashed).

**Figure 3:**
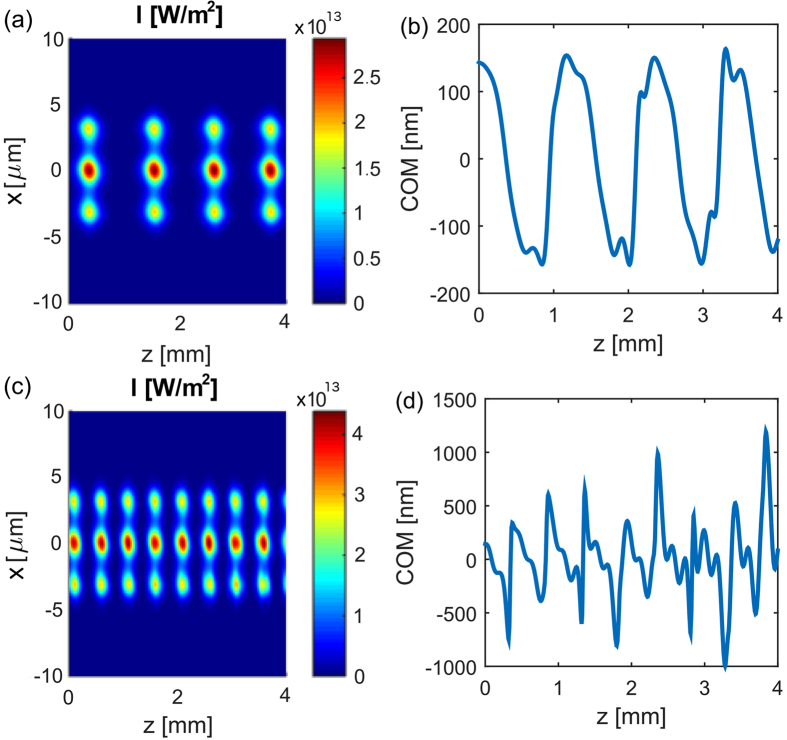
Nonlinear dynamics of light propagating in an array of three dimers described by Eq. (3) and shown in Fig. 1(a,c). The intensity distribution *I*(*x*, *z*), (**b**,**d**) the evolution of the center of mass. *ϵ*_IM_ is equal to 0.027. The input is the linear gain mode with the power density *P*_0_ equal to: (**a**,**b**) 5 · 10^6^ W/m and (**c**,**d**) 10^8^ W/m.

**Figure 4:**
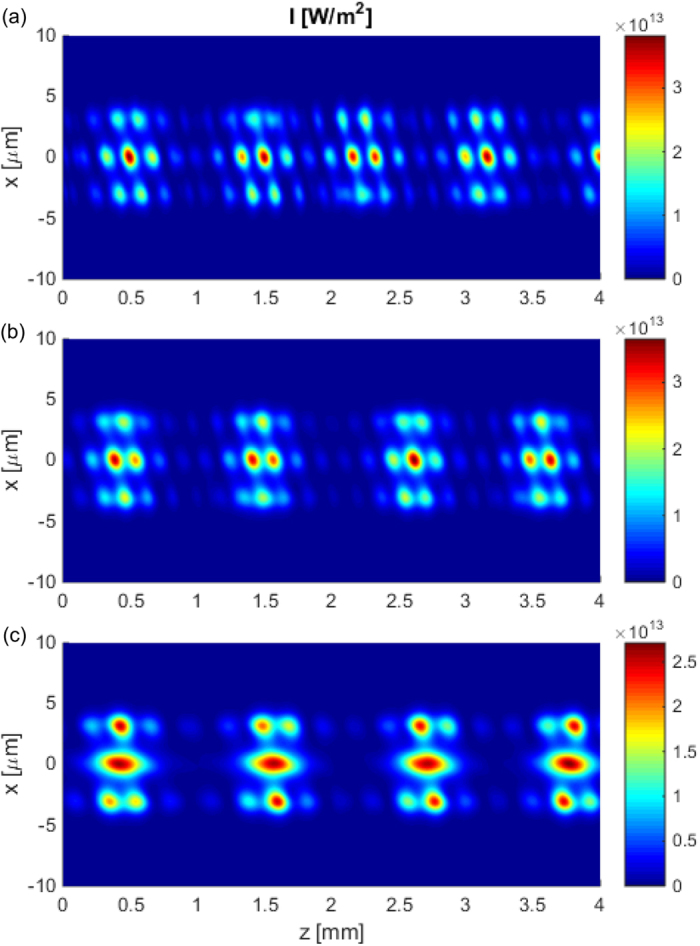
The intensity distributions *I*(*x*, *z*) showing the nonlinear dynamics of light propagating in an array built of three dimers with the same parameters as these presented in Fig. 3. The input field is in the form of (**a**) a wide Gaussian beam illuminating three dimers, (**b**) the sum of mode 1 and gain mode, (**c**) the sum of mode 2 and gain mode (for mode numbers and profiles see Fig. 2). In all the cases, the excitation power density is *P*_0_ = 5 · 10^6^ W/m.
