# Supplementary material for: Phase transition in multimode nonlinear parity-time-symmetric waveguide couplers
Source: Sci Rep. 2016 Feb 2;6:19826. doi: 10.1038/srep19826 (PMC4735683; doi:10.1038/srep19826)
Supplement: Supplementary Information [file srep19826-s1.pdf]

Supplementary materials for "Phase transition in multimode nonlinear parity-time-symmetric waveguide couplers"

Wiktor Walasik[1] and Natalia M. Litchinitser[1]

[1] *Department of Electrical Engineering, University at Buffalo, The State University of New York, Buffalo, New York 14260, USA*

(Dated: November 20, 2015)

In the main text of the manuscript, we have studied the nonlinear $\mathcal{PT}$-symmetric coupler built of three dimers. Here, we compare its $n_{\text{eff}}(\epsilon_{\text{IM}})$ diagrams and the nonlinear dynamics of light propagation with simpler structures built of one or two dimers described by Eq. (3) in the main text. The geometries of the structures described in these supplementary materials are presented in Fig. S1.

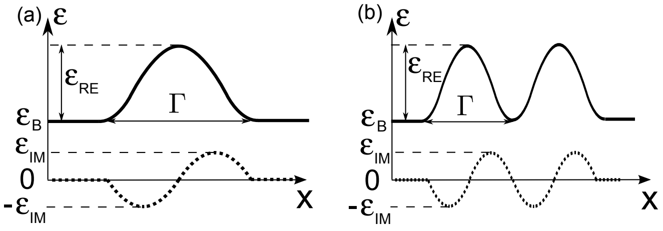

FIG. S1. Geometry of the $\mathcal{PT}$-symmetric couplers built of (a) one and (b) two cosine-like dimers. The period of the cosine-like dimers is denoted by Γ. ϵ_B denotes the background relative permittivity; ϵ_{RE} and ϵ_{IM} denote the modulation amplitude of the real and the imaginary part of relative permittivity, respectively.

Linear $n_{\mathit{eff}}(\epsilon_{IM})$ diagrams for a single multimode dimer

In the main text, we have reported a new type of $n_{\text{eff}}(\epsilon_{\text{IM}})$ diagrams for the multimode couplers built of more than one dimer. Here, we compare these new diagrams [shown in Fig. 2(a), (b) in the main text] with the $n_{\text{eff}}(\epsilon_{\text{IM}})$ curves of a multimode waveguide composed of a single $\mathcal{PT}$-symmetric dimer. Figure S2 presents the $n_{\text{eff}}(\epsilon_{\text{IM}})$ curves for a single dimer shown in Fig. S1(a). The parameters of the dimer are $\Gamma = 3$ μm, $\epsilon_B = 2$, and $\epsilon_{\text{RE}} = 0.3$. The modulation depth of the real part of permittivity is chosen in such a way that the dimer supports more than one pair of modes. In this case, the curves corresponding to higher-order modes appear below the curves of the lower-order modes, as it was shown in Ref. [1]. In contrast to the $n_{\text{eff}}(\epsilon_{\text{IM}})$ relations of a single multimode dimer presented in Fig. S2, the $n_{\text{eff}}(\epsilon_{\text{IM}})$ curves of a multimode coupler that is built of more than one dimer, as shown in Fig. 2 in the main text and in Fig. S3, present a different type of behavior. In this system, the $n_{\text{eff}}(\epsilon_{\text{IM}})$ curves of the higher-order modes appear inside the curves corresponding to the lower-order modes.

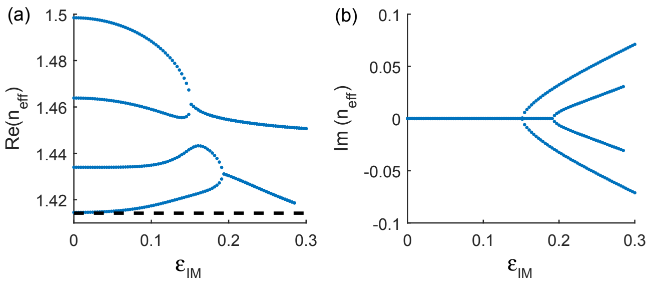

FIG. S2. $n_{\text{eff}}(\epsilon_{\text{IM}})$ relations for a single cosine-like dimer described by Eq. (3) and shown in Fig. S1(a). The (a) real part and (b) imaginary part of the effective index are shown as a function of the imaginary part of the permittivity modulation depth ϵ_{IM}. Dimer parameters (given in the text) are chosen in such a way that the dimer supports multiple modes. The black dashed line denotes the refractive index of a background medium $\sqrt{\epsilon_B}$.

Coupler built of two dimers

In the main text, we have presented the description of a nonlinear coupler described by a cosine-like permittivity profile [Eq. (3) in the main text] built of three $\mathcal{PT}$-symmetric dimers. Here, we complete the description by studying an array composed of two dimers, whose geometry is presented in Fig. S1(b). The linear $n_{\text{eff}}(\epsilon_{\text{IM}})$ diagram of the system of two dimers, presented in Fig. S3(a), (b), show qualitatively similar behavior to the coupler built of three dimers. Here, the novel type of $n_{\text{eff}}(\epsilon_{\text{IM}})$ relations is also observed, where the curves of the lower-order mode pairs enclose the curves corresponding to higher-order mode pairs. This confirms our conclusions that the $n_{\text{eff}}(\epsilon_{\text{IM}})$ curves of a multimode $\mathcal{PT}$-symmetric coupler built of a single dimer are qualitatively different from the curves of couplers built of more than one dimer.

Similar to the case of a coupler built of three dimers, here we also observe the nonlinearly-triggered-$\mathcal{PT}$ transition from the full to the broken $\mathcal{PT}$-symmetric regime. Linear modal studies of this system show that this transition occurs at $\epsilon_{\text{IM}} = 0.0278$. The nonlinear propagation of light in the system just above the $\mathcal{PT}$ transition at low and high input power levels is shown in Figs. S4(a) and (b), respectively. At the low power level, the oscillation

period is approximately equal to 1.4 mm, and the peak power corresponds to the nonlinear permittivity modulation depth of the order of $6 \cdot 10^{-4}$. For high power, the period decreases to 0.4 mm, and the maximum nonlinear permittivity modulation depth increases to $1.8 \cdot 10^{-3}$. In the case of two dimers, the effect of the increase of the amplitude of the imaginary part of permittivity is also studied. Figures S4(c), (d) present the light propagation for the same two input powers as in Figs. S4(a), (b) but in the system located higher above the $\mathcal{PT}$ transition. In this case, the oscillation period decreased to 0.25 mm. Contrary to the case of lower amplitude of the imaginary part, the increase of the initial power for the case presented in Figs. S4(c) and (d) does not modify the oscillation period, and it is the same for both power levels. Only the initial propagation distance required to initiate the oscillations (from the input plane $z = 0$ to the first intensity maximum) is reduced with the increase of the excitation power because the same intensity level is reached earlier by the system, where more power was injected.

Finally, we have studied the behavior of the coupler built of two $\mathcal{PT}$-symmetric dimers when more than one mode of the system is excited. The field profiles of the linear modes supported by the system are presented in Fig. S3(c), (d). Figure S5 presents the evolution of the field in the case when the gain mode and the fundamental mode are excited simultaneously. The interference pattern obtained here resembles these for the coupler built of three waveguides presented in Fig. 4 in the main text. However, for the simpler system composed of two waveguides, the interference pattern is less complex than for the coupler built of three $\mathcal{PT}$-symmetric waveguides.

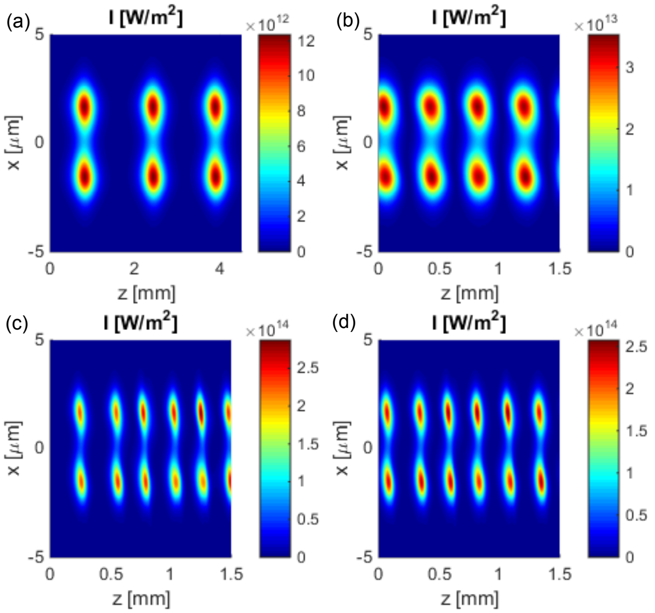

FIG. S4. The intensity distributions $I(x, z)$ showing the nonlinear dynamics of light propagating in an array built of two dimers described by Eq. (3). The geometry of the structure is shown in Fig. S1 and in the inset of supblot (a). The parameters are: $\Gamma = 3$ μm, $\epsilon_{\rm RE} = 0.05$, $\alpha = 10^{-19}$ m^2/V^2 and (a), (b) $\epsilon_{\rm IM} = 0.028$, (c), (d) $\epsilon_{\rm IM} = 0.030$. Excitation power density P_0 is equal to: (a), (c) 10^5 W/m and (b), (d) 10^8 W/m. The propagation distance shown in (a) is three times longer than in (b)–(d).

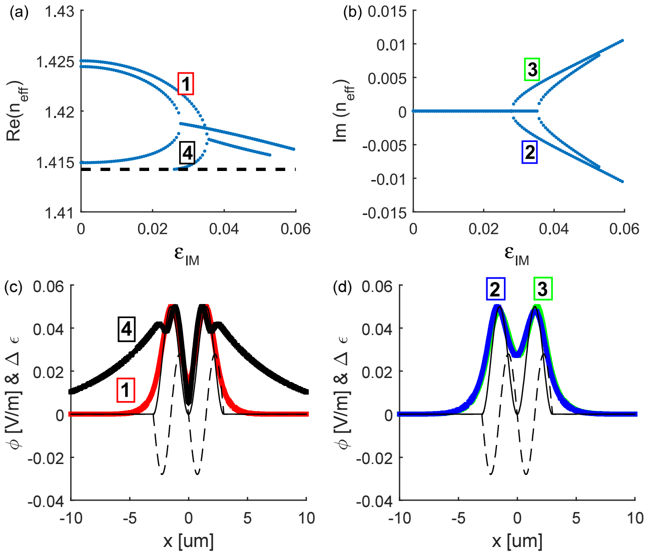

FIG. S3. (a), (b) $n_{\rm eff}(\epsilon_{\rm IM})$ relations for a $\mathcal{PT}$-symmetric coupler composed of two dimers; (a) the real and (b) the imaginary part of the effective index of the modes as a function of the imaginary part of permittivity $\epsilon_{\rm IM}$ in the linear case. The black dashed line denotes the refractive index of a background medium $\sqrt{\epsilon_B}$. (c), (d) Absolute value of the field distributions $|\phi(x)|$ of (c) the lossless modes [mode 1 (fundamental)—red, mode 4 (lowest effective index)—black], (d) the coupled modes with loss (blue) and gain (green) at $\epsilon_{\rm IM} = 0.028$. Black curves indicate $\Re e\{\Delta\epsilon\}$ (solid) and $\Im m\{\Delta\epsilon\}$ (dashed).

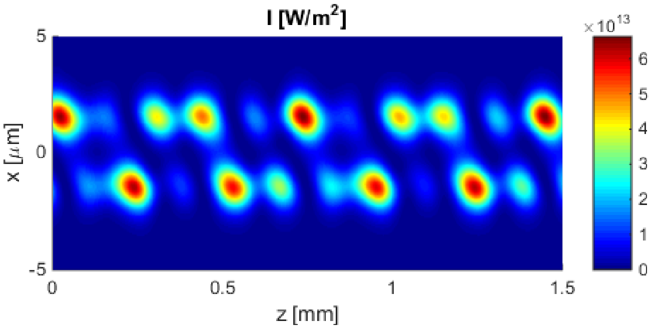

FIG. S5. The intensity distributions $I(x, z)$ showing the nonlinear dynamics of light propagating in an array built of two dimers with the same parameters as these presented in Fig. S4(b). The input field is in the form of the sum of mode 1 and gain mode. The excitation power density is $P_0 = 10^8$ W/m [the same as in Fig. S4(b)].

[1] C. Huang, F. Ye, and X. Chen, Phys. Rev. A **90**, 043833 (2014).
